# Immunohistochemical comparison of CD5, lambda, and kappa expression in primary and recurrent buccal Mucosa-associated lymphoid tissue (MALT) lymphomas

**DOI:** 10.1186/1746-1596-6-82

**Published:** 2011-09-06

**Authors:** Toshiaki Tanaka, Kenichirou Kitabatake, Mituyoshi Iino, Kaoru Goto

**Affiliations:** 1 Department of Anatomy and Cell Biology, School of Medicine, Yamagata University, 2-2-2 Iidanishi, Yamagata 990-9585, Japan; 2 Department of Dentistry, Oral and Maxillofacial · Plastic and Reconstructive Surgery, School of Medicine, Yamagata University, 2-2-2 Iidanishi, Yamagata 990-9585, Japan; 3 Density and Oral surgery, Shinjyo Tokusyukai Hospital, 4623 Torigoeazakomaba, Shinjyo 996-0041, Japan

## Abstract

Mucosa-associated lymphoid tissue (MALT) lymphoma is a type of extranodal marginal zone B-cell lymphoma and is a distinct subtype of non-Hodgkin's lymphoma.

Primary MALT lymphomas can also occur in the oral cavity, although their appearance in this location is rare. The neoplastic cells of which MALT lymphomas are composed express B-cell antigens and show monotypic immunoglobulin expression with light-chain restriction.

Although neoplastic MALT lymphoma cells do not express CD5, previous studies have shown that CD5 positive MALT lymphomas are more prone to dissemination than those that do not express CD5. Moreover, there are some reports that describe kappa- and lambda- dual light chain expression in B cell malignant neoplasms.

A 66-year-old Japanese woman with swelling of the right buccal mucosa was referred to our hospital. The lesion was excised and was pathologically diagnosed as a MALT lymphoma tumor with a t(11;18)(q21;q21) chromosome translocation.

Swelling of the right buccal mucosa recurred 2 years later. The recurrent tumor was then excised and pathologically diagnosed as MALT lymphoma.

Immunohistochemical examination of CD5, lambda, and kappa expressions revealed that the primary tumor was positive for CD5, kappa, and lambda, but the recurrent tumor was weakly positive for CD5 and kappa.

With respect to lambda positivity, the recurrent tumor showed negativity.

Our study suggests that immunohistochemical expression of CD5, kappa, and lambda in oral MALT lymphoma have the risk of recurrence.

We first described the recurrence of CD5 positive MALT lymphoma in the oral cavity and compared the immunohistochemical expressions of CD5, lambda, and kappa between the primary and recurrent tumors.

## Background

Mucosa-associated lymphoid tissue (MALT) lymphoma is a type of extranodal marginal zone B-cell lymphoma and is a distinct subtype of non-Hodgkin's lymphoma [1].

The most common site of MALT lymphomas is the stomach, and the majority of gastric MALT lymphomas are associated with *Helicobacter pylori *infection [2]. Other sites where MALT lymphomas can occur include the orbit, lung, salivary glands, thyroid, skin, intestine, and liver [3]. Salivary gland and thyroid MALT lymphomas are associated with autoimmune disorders such as Sjogren's syndrome and Hashimoto disease, respectively [4,5]. Primary MALT lymphomas can also occur in the oral cavity, although their appearance in this location is rare [6].

The neoplastic cells of which MALT lymphomas are composed express B-cell antigens and show monotypic immunoglobulin expression with light-chain restriction [7,8].

Typically, neoplastic MALT lymphoma cells do not express CD5 [8] although some researchers have reported that CD5 positive MALT lymphomas exist [7]. Furthermore, previous studies have shown that CD5 positive MALT lymphomas are more prone to dissemination than those that do not express CD5 [9].

Demonstration of light chain restriction in a B lymphocyte population is considered proof of monoclonality and indicates malignancy [10]. Moreover, there are some reports that describe kappa- and lambda- dual light chain expression in B cell malignant neoplasms [10,11].

In oral MALT lymphoma, there are no reports that investigate immunohistochemical comparison of CD5, lambda, and kappa expression in primary and recurrent MALT lymphomas.

We studied the case of a patient with a recurrent CD5 positive MALT lymphoma located in the buccal mucosa that showed weak, positive expression of CD5 and kappa and negative expression of lambda.

## Case presentation

### Clinical presentation

A 66-year-old Japanese woman with swelling of the right buccal mucosa was referred to our hospital. She was generally healthy and her medical, dental, and family histories were normal. Routine laboratory examinations did not reveal any abnormalities.

The lesion was excised and was pathologically diagnosed as a MALT lymphoma tumor with a t(11;18)(q21;q21) chromosome translocation [12]. Swelling of the right buccal mucosa recurred 2 years later. Clinical examination at that time revealed a sessile, hard, elastic, and movable mass in the right buccal mucosa; similar to previous clinical findings, this mass was covered with normal mucosa.

The recurrent tumor was excised under general anesthesia, and general safety margins were maintained. The recurrent tumor was similar to the primary tumor; a sessile, white, elastic, and circumscribed mass. The recurrent tumor was well demarcated and was covered with normal mucosa. The length of the recurrent tumor was 3.3 cm.

To determine whether the lymphoma had spread to other sites, computed tomography (CT) scans and magnetic resonance imaging (MRI) of the whole body were performed and endoscopic examination of the stomach was added. All test results were negative for the presence of lymphoma in the other sites. Clinically, the patient remained in good general health, and evidence of tumor reactivation 6 months after the treatment was lacking.

### Microscopic findings

**Figure 1:**
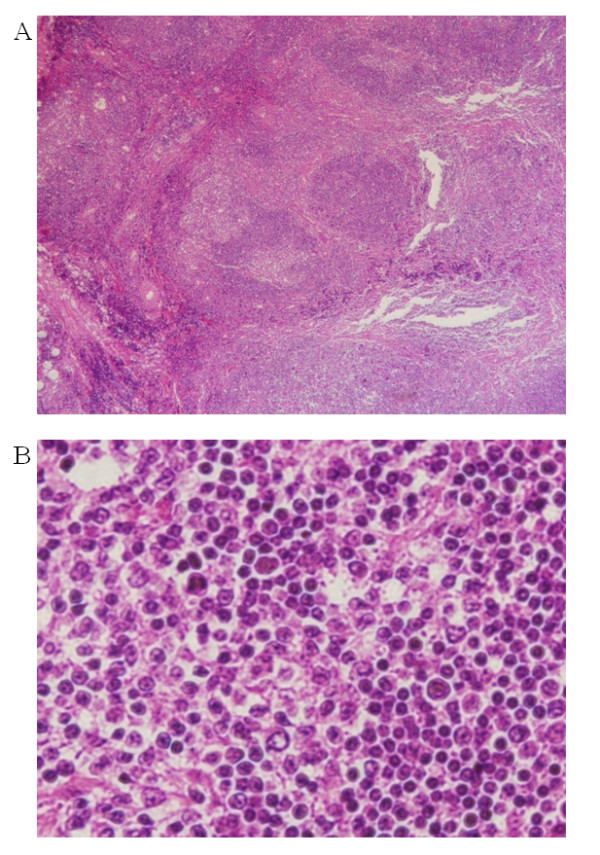
**Light microscopic examination of primary tumor**. Low-power magnification (×50) show some colonized lymphoid follicles with mantle zone in the primary MALT lymphoma (A). High-power magnification (×630) show centrocyte-like cells, monocytoid B-cells, and neoplastic cells which have Dutcher body in the primary MALT lymphoma (B).

**Figure 2:**
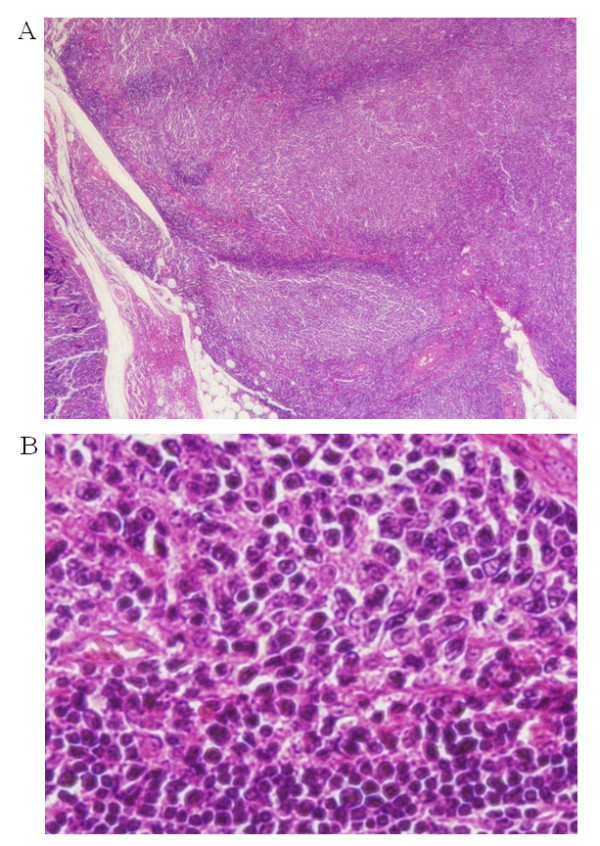
**Light microscopic examination of recurrent tumor**. Low-power magnification (×50) show that recurrent tumor cells formed follicular colonization in the germinal centers throughout the tumor (A). High-power magnification (×630) show centrocyte-like cells and plasmacytoid differentiation in the recurrent MALT lymphoma. Plasmacytoid differentiation was more prevalent in the recurrent tumor than in the primary tumor (B).

Paraffin sections of the primary tumor revealed (under a low-power field) some colonized lymphoid follicles with a mantle zone and diffuse proliferation of tumor cells in the marginal zone (Figure 1A). On the other hand, paraffin sections of the recurrent tumor revealed that the tumor cells showed follicular colonization in the germinal centers throughout the tumor (Figure 2A).

Under high-power fields, small- or medium-sized centrocyte-like cells with indented nuclei and moderate cytoplasm volume were found in the primary and recurrent tumor (Figure 1B and 2B). Monocytoid B-cells with round nuclei and abundant, pale cytoplasm and neoplastic cells with Dutcher bodies were more abundant in the primary tumor than in the recurrent tumor (Figure 1B and 2B).

In addition, plasmacytoid differentiation was more prevalent in the recurrent tumor than in the primary tumor (Figure 1B and 2B).

### Immunohistochemical findings

For immunohistochemical analyses, paraffin sections were incubated with primary antibodies, including CD10 (M0727, DAKO, Copenhagen, Denmark), CD20 (M0755, DAKO), CD43 (MT1 clone, Leica Microsystems, UK), CD79a (JCB117 clone, Dakocytomation, Denmark), Bcl-2 (M0887, DAKO), Bcl-6 (M7211, Dakocytomation), IgG (A0423, DAKO), IgG4 (05-3800, Nichirei-Zymed, Tokyo, Japan), IgM (A0425, Dakocytomation), Lambda (A0193, DAKO), Kappa (A0191, DAKO), cyclin D1 (SP4 clone, DBIOSYS, Washington D.C, U.S.A), CK (AE1/3) (M0835, DAKO), and *Helicobacter pylori *(B0471, Dakocytomation).

The sites for antigen-antibody reactions were visualized using a standard avidin-biotinylated-peroxidase complex method. The primary tumor cells were positive for bcl-2, CD20, CD43, CD79a, Lambda, Kappa and was negative for CD10, IgG, IgG4, IgM, cyclin D1, bcl-6, CK (AE1/3), and *Helicobacter pylori *(data not shown) [12]. The overall staining results of the recurrent tumor were similar to those of the initial tumor. The recurrent tumor showed negativity for CD10, IgG, IgG4, IgM, cyclin D1, Bcl-6, CK (AE1/3), and *Helicobacter pylori *(data not shown) except that the recurrent tumor showed negativity for lambda and weak positivity for kappa.

A previous study revealed that MALT lymphomas that express CD5 tend to recur and disseminate [9]. To ascertain whether the primary tumor expressed CD5, the tumor specimen was incubated with a CD5 antibody. Moreover, expressions of lambda and kappa in the primary tumor specimen were compared with those in the recurrent tumor specimen.

**Figure 3:**
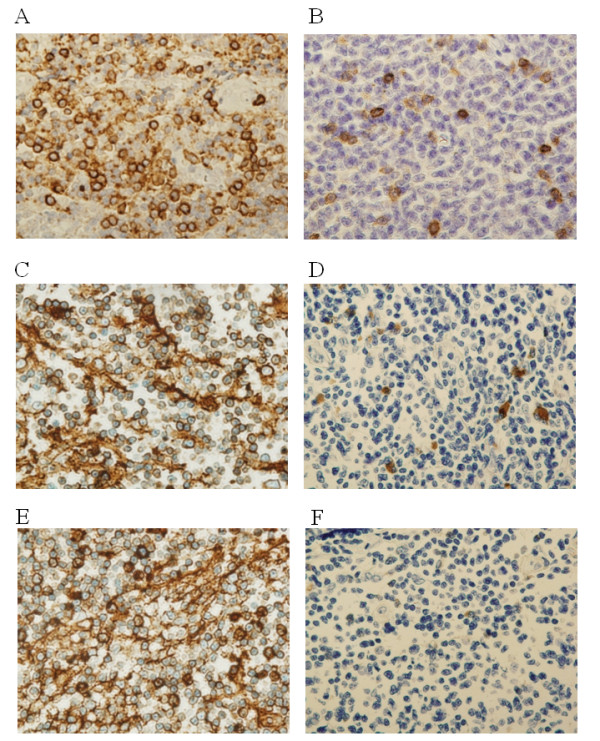
**Immunohistochemical comparison of CD5, lambda, and kappa expression in primary and recurrent buccal MALT lymphomas (magnification ×630)**. Immunohistochemical staining of primary tumor sections show that the tumor cells are positive for CD5 (A), kappa (C), and lambda (E). Immunohistochemical staining of recurrent tumor sections show that the tumor cells are weakly positive for CD5 (B) and kappa (D) and negative for lambda (F).

Immunohistochemical examination of CD5, lambda, and kappa expressions revealed that the primary tumor was positive for CD5 (Figure 3A), kappa (Figure 3C), and lambda (Figure 3E), but the recurrent tumor was weakly positive for CD5 (Figure 3B) and kappa (Figure 3D).

With respect to lambda positivity, the recurrent tumor showed negativity (Figure 3F).

## Discussion

Immunohistochemically, malignant MALT lymphoma cells express markers of B-cell lineage, but are distinct from follicular lymphomas (which express CD10), mantle cell lymphomas (which express cyclin D1 and CD5), and small lymphocytic lymphomas (which express CD5 and CD23) [13].

Typically, the neoplastic MALT lymphoma cells coexpress CD20+ and surface light-chain restriction but are negative for CD10 and CD5. Some researchers have documented expression of CD5 in MALT lymphomas, which is rare. Aberrant CD5-expression is thought to be a marker of early dissemination and aggressive behavior in some patients, but CD5 expression in localized, indolent MALT-type lymphomas has been reported [13].

CD5 is a T-cell antigen that is expressed on normal B-cells, in cord blood, adult peripheral blood, spleen, and lymph nodes. CD5 is also occasionally expressed on B-cell neoplasms, but its clinicopathological significance in low-grade B-cell non-Hodgkin's lymphomas (NHL), notably MALT lymphomas, remains obscure [7]. In the oral cavity, previous report described that primary MALT lymphoma with the expression of CD5 occurred in the tongue [14]. Although MALT lymphoma expressed CD5, this MALT lymphoma did not recur. However, we experienced that CD5 positive expression of MALT lymphoma in buccal mucosa occurred recurrence. Whether CD5 expression is relevant to the prognosis of patients with MALT lymphoma is controversial [9].

Kojima and colleagues reported that MALT lymphoma occurred in the soft palate and recurred [6,15]. This MALT lymphoma showed positivity for kappa but negativity for CD5.

Compared with our primary case and Kojima and colleagues' case, our primary case expressed CD5, kappa-, and lambda-light chain. The presence of both kappa- and lambda-light chain-restricted B-cell populations in B-cell lymphomas, including MALT lymphoma, is an unusual findings [16].

Edinger *et al. *reported 2 other examples of primary cutaneous marginal zone lymphomas that express both kappa- and lambda-light chain-restricted B-cell populations [17].

In this study, kappa- and lambda-light chains were clonally related to each other [16]. Fujiwara *et al. *described the aggressive B cell lymphoma with kappa- and lambda- dual light chain expression [11]. They considered that kappa- and lambda- dual light chain expression was involved in the aggressiveness of B cell lymphoma.

Harby *et al. *observed that the frequent lambda- light chain gene rearranged and expressed in murine CD5+ B lymphoma cells and these lymphoma cells had a functional kappa- chain allele when induced for kappa- chain expression with bacterial lipopolysaccharide [18]. This mechanism in CD5+ B lymphoma cells may be related to the recurrence of our case.

Gastric MALT lymphomas are correlated with *Helicobacter pylori *gastritis because colonization of *Helicobacter pylori *in the gastric mucosa results in lymphoid infiltration and the formation of acquired lymphoid tissue [19].

Moreover, T cells are specific for *Helicobacter pylori *and neoplastic B cells in gastric MALT lymphomas generate autoantibodies [20].

We considered that the pathogenesis of oral MALT lymphoma is related to *Helicobacter pylori *infection; however, *Helicobacter pylori *were not detected by immunohistochemical analysis or endoscopy in either the primary or the recurrent tumors. These results suggest that oral MALT lymphoma etiology is a result of several distinct factors and not exclusively *Helicobacter pylori *infection.

Although it is well known that MALT lymphomas frequently occur in the background of inflammatory disorders when B cell clones become independent in their growth [14], the development of oral MALT lymphoma is largely unknown. Present studies are considering allergies to metals and periodontitis as factors for the development of MALT lymphoma of the oral cavity. Therefore, further investigations are necessary to clarify the mechanism of the development of oral MALT lymphoma.

Our study suggests that immunohistochemical expression of CD5, kappa, and lambda in oral MALT lymphoma have the risk of recurrence. Our study raises the possibility to decide the therapeutic strategy whether oral MALT lymphoma expresses CD5, kappa, and lambda.

Although the present patient had no recurrence over 6 months after excision of the recurrent tumor, careful observation is needed, because the clinicopathological significance of MALT lymphomas with this rare phenotype remains obscure [7].

We first described the recurrence of CD5 positive MALT lymphoma in the oral cavity and compared the immunohistochemical expressions of CD5, lambda, and kappa between the primary and recurrent tumors.

## Conclusions

We studied the case of a patient with a recurrent CD5 positive buccal MALT lymphoma in the oral cavity and compared the immunohistochemical expressions of CD5, lambda, and kappa between the primary and recurrent tumors. We first described the recurrence of CD5, lambda, and kappa positive MALT lymphoma in the oral cavity and recurrent MALT lymphoma showed weak positivity for CD5 and kappa and negativity for lambda.

## Consent

Written informed consent was obtained from the patient for publication of this case report and any accompanying images. A copy of the consent is available for review by the Editor-in-chief of the journal.

## List of abbreviations

MALT: mucosa-associated lymphoid tissue; IgG: immunoglobulin G; IgM: immunoglobulin M; NHL: non-Hodgkin's lymphomas; CT: computed tomography; MRI: magnetic resonance imaging.

## Competing interests

The authors declare that they have no competing interests.

## Authors' contributions

TT analyzed the data and wrote the manuscript as a major contributor. KK contributed to management of the patient. IM participated in study design and coordination. GK carried out the histopathological evaluation and helped to write manuscript. All authors have read and approved the final manuscript.
